# PACAP/Receptor System in Urinary Bladder Dysfunction and Pelvic Pain Following Urinary Bladder Inflammation or Stress

**DOI:** 10.3389/fnsys.2017.00090

**Published:** 2017-12-04

**Authors:** Beatrice M. Girard, Katharine Tooke, Margaret A. Vizzard

**Affiliations:** Department of Neurological Sciences, Larner College of Medicine, The University of Vermont, Burlington, VT, United States

**Keywords:** micturition, neuropeptides, inflammation, psychogenic stress, NGF, cyclophosphamide, PAC1, PACAP(6-38)

## Abstract

Complex organization of CNS and PNS pathways is necessary for the coordinated and reciprocal functions of the urinary bladder, urethra and urethral sphincters. Injury, inflammation, psychogenic stress or diseases that affect these nerve pathways and target organs can produce lower urinary tract (LUT) dysfunction. Numerous neuropeptide/receptor systems are expressed in the neural pathways of the LUT and non-neural components of the LUT (e.g., urothelium) also express peptides. One such neuropeptide receptor system, pituitary adenylate cyclase-activating polypeptide (PACAP; *Adcyap1*) and its cognate receptor, PAC1 (*Adcyap1r1*), have tissue-specific distributions in the LUT. Mice with a genetic deletion of PACAP exhibit bladder dysfunction and altered somatic sensation. PACAP and associated receptors are expressed in the LUT and exhibit neuroplastic changes with neural injury, inflammation, and diseases of the LUT as well as psychogenic stress. Blockade of the PACAP/PAC1 receptor system reduces voiding frequency in preclinical animal models and transgenic mouse models that mirror some clinical symptoms of bladder dysfunction. A change in the balance of the expression and resulting function of the PACAP/receptor system in CNS and PNS bladder reflex pathways may underlie LUT dysfunction including symptoms of urinary urgency, increased voiding frequency, and visceral pain. The PACAP/receptor system in micturition pathways may represent a potential target for therapeutic intervention to reduce LUT dysfunction.

## Introduction

Micturition, the expulsion of urine from the urinary bladder through the urethra, is an important part of everyday life. Micturition is a basic physiological function to which we give little thought until this daily behavior is changed in some way. Normal micturition involves the filling and storage of urine in the bladder, and the periodic voiding of urine at socially appropriate times. Storage and elimination functions involve the reciprocal functions of the bladder, urethra and external urethral sphincter (i.e., urethral rhabdosphincter). These two modes of operation are controlled by the coordination of the structural features of the bladder and complex neural pathways organized in the CNS and PNS (Elbadawi, 1996; Andersson, 2004).

## Anatomy of the Lower Urinary Tract (LUT)

The LUT consists of the urinary bladder and urethra. The urethra is composed of both smooth and striated muscle (Andersson, 2004). The urinary bladder is a hollow, smooth muscle organ and consists of two main parts, the bladder body and the bladder base (Elbadawi, 1996; Andersson, 2004). The bladder body is located above the opening of the urethra. The base contains the trigone, urethrovesical junction, and the anterior bladder wall (Elbadawi, 1996; Andersson, 2004). The urinary bladder wall is organized into layers: mucosa, muscle, and serosa and adventitia (Tank, 2009). The mucosal layer consists of transitional epithelial cells that line the lumen of the bladder and a lamina propria beneath the basement membrane of the epithelial cells (Tank, 2009). The urothelium, a layer of epithelial cells, lines the lumen of the bladder. The lamina propria is composed of a thick layer of cells (e.g., fibroblasts, adipocytes, interstitial cells, and afferent and efferent nerve terminals), collagen, elastic fibers and blood vessels (Andersson and McCloskey, 2014). The muscular layer is composed of smooth muscle cells that constitute the muscular wall of the bladder. It is structurally different from the smooth muscle of the trigone and urethra because it consists of an inner and outer longitudinal layer and a middle circular layer of smooth muscle (Andersson, 2004). These muscle cells relax and elongate during bladder filling, whereas the urethra is closed and non-compliant. Bladder emptying involves the coordinated contraction of the detrusor muscle as well as relaxation, opening, and dilation of the urethra (Elbadawi, 1996; Andersson, 2004). The serosal layer surrounds the superior and lateral external surfaces of the bladder wall, whereas loose connective tissue, the adventitia, surrounds the retroperitoneal bladder wall (Tank, 2009).

## Urothelium

The urothelium is a layer of transitional epithelium capable of detecting diverse stimuli including, mechanical, chemical, and thermal stimuli. The urothelium is composed of three layers: an innermost basal cell layer attached to a basement membrane, an intermediate layer, and a superficial apical layer (Birder, 2005; Apodaca et al., 2007; Birder and de Groat, 2007; Birder et al., 2009, 2014; Birder and Andersson, 2013). The apical layer contains large, hexagonal shaped umbrella cells that change shape during filling to expand the epithelial surface giving the transitional epithelium its name. The apical layer also acts as a barrier against substances in the urine that may be detrimental to the bladder. This barrier function can be compromised during injury or inflammation, allowing toxic substances to reach the suburothelial nerve plexus and muscular layers, contributing to urinary urgency, frequency, and pain during voiding (Birder, 2005; Apodaca et al., 2007; Birder and de Groat, 2007; Birder et al., 2009, 2014; Birder and Andersson, 2013).

The urothelium, once thought to act only as a passive barrier, is now appreciated to play important and active roles in afferent signaling. This active function involves receiving afferent nerve input from nearby nerves in the suburothelial nerve plexus and in response, communicating directly with the nerves that innervate the bladder, the smooth muscle of the bladder and local inflammatory cells (Birder, 2005; Apodaca et al., 2007; Birder and de Groat, 2007; Birder et al., 2009, 2014; Birder and Andersson, 2013). The apical layer of the urothelium expresses surface receptors and ion channels (Birder, 2005; Birder and de Groat, 2007; Birder and Andersson, 2013; Merrill et al., 2016) enabling the recognition of diverse sensory stimuli. Receptors found in the urothelium are numerous and diverse and include: B1 and B2 bradykinin receptors activated by bradykinin (Chopra et al., 2005; Birder and de Groat, 2007), p75^NTR^, TrkA, and TrkB activated by neurotrophins (e.g., NGF, BDNF) (Qiao and Vizzard, 2002b; Murray et al., 2004; Petruska and Mendell, 2004; Merrill et al., 2016), purinergic receptors (P2X and P2Y) activated by ATP (Cockayne et al., 2005; Wang et al., 2005; Ford and Cockayne, 2011; Birder and Andersson, 2013), adrenergic receptors activated by norepinephrine (Birder and Andersson, 2013), cholinergic receptors activated by acetylcholine (Beckel et al., 2006; Birder and Andersson, 2013), neuropeptide receptors including PACAP type 1 receptor (PAC1) and VIP receptor 2 (VPAC2) (Arms and Vizzard, 2011; Merrill et al., 2013a; Gonzalez et al., 2014b). Transient receptor potential (TRP) channels, activated by inflammatory mediators, temperature changes and/or pH changes within the bladder (Birder and Andersson, 2013; Merrill et al., 2016), are also expressed by the urothelium, including members of the vanilloid (V), melastatin (M), and ankyrin (A) families: TRPV1, TRPV2, TRPV4, TRPM8, and TRPA1 (Skryma et al., 2011; Merrill et al., 2012; Avelino et al., 2013; Gonzalez et al., 2014b). In addition, recent studies demonstrate the presence of another cell type in the rat urethra that recognizes and responds to diverse stimuli. Cholinergic chemosensory cells are found in the rat urethra in close proximity to sensory nerve fibers expressing nicotinic acetylcholine (ACh) receptors (Deckmann et al., 2014). These cells respond to bitter (denatonium), umami (monosodium glutamate) and uropathogenic *Escherichia coli* stimulation and intraurethral denatonium increases detrusor activity (Deckmann et al., 2014). It is suggested that these chemosensory cells in the rat urethra may monitor for the presence of hazardous compounds, release ACh and affect bladder function (Deckmann et al., 2014).

Following stimulation, the urothelium can release signaling mediators to produce localized vascular changes (Birder and de Groat, 2007; Fowler et al., 2008) and to influence adjacent tissues and cells, including: detrusor smooth muscle, afferent nerve fibers in the suburothelial nerve plexus, inflammatory cells and interstitial cells within the bladder (Birder and de Groat, 2007; Fowler et al., 2008; Birder and Andersson, 2013; Merrill et al., 2016). For example, the urothelium can release many signaling molecules, including, ATP, (Ferguson et al., 1997; Birder and Andersson, 2013), NO (Birder and Andersson, 2013), acetylcholine (Birder and de Groat, 2007), substance P, cytokines, chemokines and prostaglandins as well as a variety of neurotrophic factors (Arms and Vizzard, 2011; Merrill et al., 2013a; Gonzalez et al., 2014a,b; Merrill et al., 2016). The release of signaling molecules from the urothelium can be altered with injury, inflammation and disease (Birder, 2005; Birder and de Groat, 2007; Arms and Vizzard, 2011; Birder and Andersson, 2013; Merrill et al., 2013a, 2016; Gonzalez et al., 2014a,b).

## Neural Control of Micturition

**FIGURE 1:**
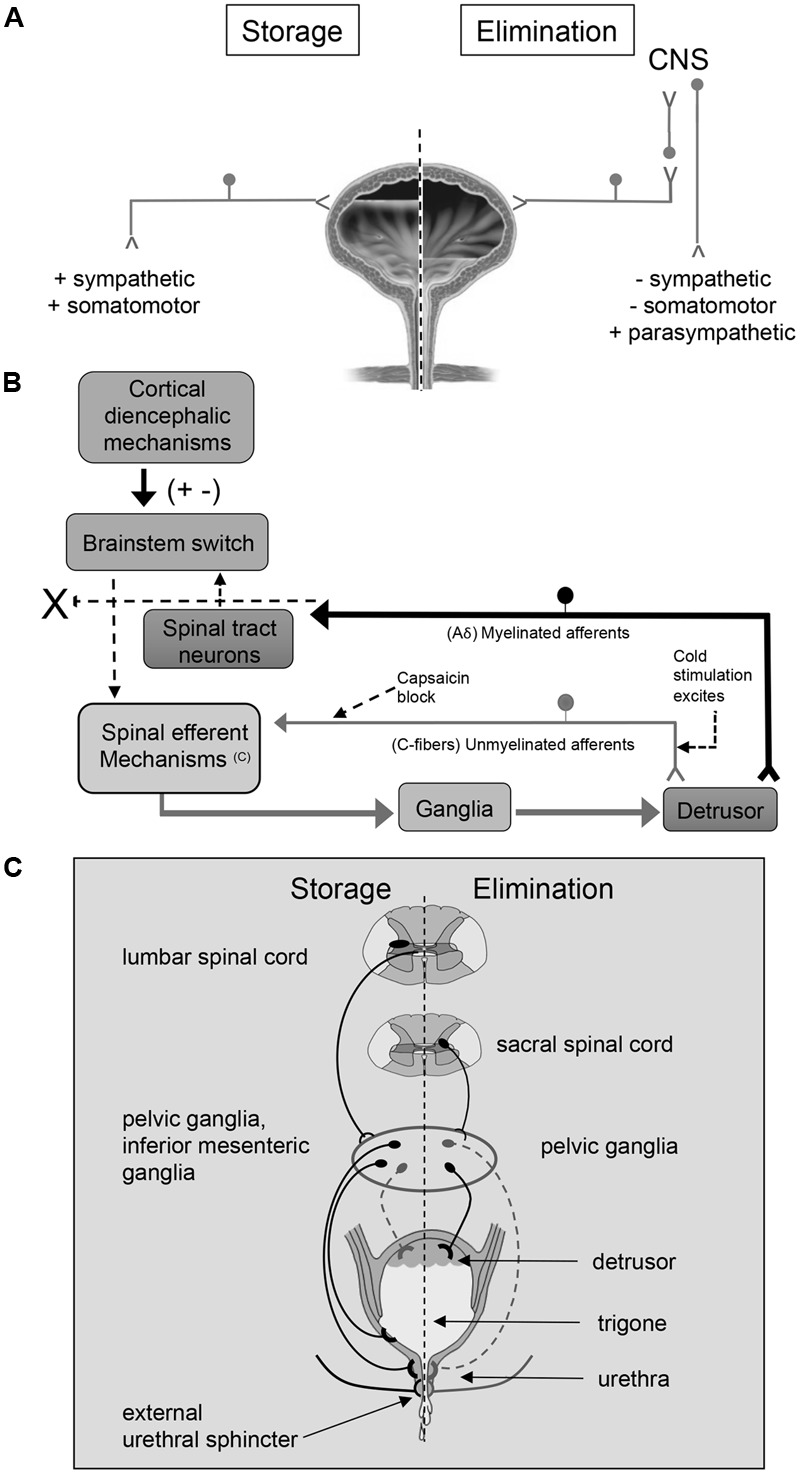
An overview of micturition reflex control. Neural control of lower urinary tract function **(A)** involves the coordinated activity of afferent **(B)** and efferent pathways **(C)**. **(B)** Micturition is initiated by a supraspinal reflex pathway that passes through a center in the brainstem [figure from (Fowler et al., 2008) with permission]. The pathway is triggered by myelinated afferents (Aδ-fibers), which are connected to the mechanoreceptors in the bladder wall. Suprasacral spinal cord injury interrupts the connections between the brain and spinal cord and initially causing the micturition reflex to be areflexic. Following SCI, a spinal micturition reflex emerges that is now triggered by unmyelinated bladder afferents (C-fibers). The C-fiber reflex pathway is usually weak or undetectable in spinal cord intact animals. Cold water stimulation of the C-fiber bladder afferents activates voiding responses in patients with SCI. The C-fiber neurotoxin, capsaicin (20–30 mg subcutaneously), blocks the C-fiber reflex in cats with SCI but does not block micturition reflexes in spinal intact cats. Intravesical capsaicin also suppresses detrusor hyperreflexia and cold-evoked reflexes in patients with neurogenic bladder dysfunction (Fowler et al., 2008). This schematic is based on results from electrophysiological studies in cats (de Groat and Yoshimura, 2006). Additional species differences in afferent control mechanisms of micturition reflexes are described in the text. **(C)** Excitatory (black) and inhibitory (gray) nerves activated during storage and elimination (voiding) are shown. External urethral sphincter (i.e., urethral rhabdosphincter) innervation via pudendal nerves is also indicated. Broken lines indicate neuronal pathways that are less well understood. Storage reflexes, activated during bladder filling, are organized primarily in the spinal cord, whereas voiding is mediated by reflex mechanisms organized in the brain. During bladder filling and storage, the sympathetic nervous system plays a major role. Preganglionic neurons in the rostral lumbar spinal cord excite sympathetic neurons in the inferior mesenteric ganglia and pelvic ganglia causing contraction of smooth muscle in the trigone and urethra, and this is coordinated with contraction of the external urethral sphincter. During bladder filling and storage, the sympathetic nervous system acts to inhibit the detrusor muscle causing relaxation and to excite the bladder neck and urethra causing contraction, preventing incontinence. However, the contribution of the sympathetic system to continence across species is debatable (broken lines). See text for additional details. During bladder filling, the parasympathetic efferent pathway to the bladder, including a population of CNS (e.g., pontine micturition center) neurons, is turned off. When a threshold level of bladder distension is reached during filling, the afferent activity from bladder mechanoreceptors switches the pathway from the storage to the elimination mode. The parasympathetic nervous system predominates during elimination (voiding). Activation of sacral preganglionic neurons excites parasympathetic ganglion neurons in the pelvic ganglia. During elimination, the activity of the parasympathetic nervous system results in urinary bladder contraction, whereas sympathetic activity and somatomotor activity is withdrawn. Figure adapted from (de Groat, 1990, 1993; de Groat et al., 1998; de Groat and Araki, 1999; Middleton and Keast, 2004; Vizzard, 2006; de Groat and Yoshimura, 2012; de Groat and Wickens, 2013; Merrill et al., 2016).

The LUT has two phases of operation (storage and elimination), that are under CNS and voluntary control (Fowler et al., 2008; Griffiths, 2015; Miyazato et al., 2017) (**Figure 1**). During the filling phase, the detrusor smooth muscle is relaxed and the urethral sphincter is contracted. In the emptying phase, the opposite occurs (Fowler et al., 2008; Griffiths, 2015; Miyazato et al., 2017). These processes are controlled by both the autonomic (sympathetic and parasympathetic) and the somatic nervous system (**Figure 1A**).

The cell bodies of the preganglionic sympathetic innervation to the urogenital organs are located in the intermediolateral gray matter of the lower thoracic and upper lumbar spinal cord (T10-L2) (de Groat, 1990, 1993; Elbadawi, 1996; Holstege, 2005; Fowler et al., 2008). After projecting from the spinal cord, these cells form lumbar splanchnic nerves and synapse in the inferior mesenteric ganglion. Postganglionic sympathetic fibers then travel from the ganglion to urogenital organs through the pelvic and hypogastric nerves (de Groat, 1990, 1993; Elbadawi, 1996; Holstege, 2005; Fowler et al., 2008) (**Figure 1C**). These sympathetic fibers act either directly on the bladder or indirectly via connections with vesical or pelvic ganglia (de Groat, 1990, 1993; Elbadawi, 1996; Holstege, 2005; Fowler et al., 2008). The sympathetic system is activated alone during bladder filling, and acts to inhibit the detrusor muscle causing relaxation and to excite the bladder neck and urethra causing contraction (de Groat, 1990, 1993; Elbadawi, 1996; Holstege, 2005; Fowler et al., 2008) (**Figure 1C**). However, the contribution of the sympathetic system to continence across species is debatable. For example, in humans and cats, sympathetic innervation of the detrusor may contribute to continence (Vaughan and Satchell, 1992; Khadra et al., 1995; Andersson and Arner, 2004) by modulating transmission in parasympathetic ganglion neurons (De Groat and Saum, 1972), but this is less clear in rodents (Keast et al., 2015). There is limited evidence that sympathetic noradrenergic pelvic ganglion neurons innervate the bladder, which parallels the observation of sparse sympathetic noradrenergic innervation of the detrusor in rodents (Andersson and Wein, 2004; Forrest et al., 2014).

The cell bodies of the preganglionic parasympathetic nerves that innervate the bladder are located in the lateral horn of sacral spinal cord (S2–S4). These fibers project from the spinal cord in ventral roots and immediately separate as pelvic splanchnic nerves (i.e., pelvic nerve). The pelvic nerve synapses on terminal ganglia (e.g., intramural ganglia, MPG) and innervates the urogenital organs (de Groat, 1990, 1993; Elbadawi, 1996; Holstege, 2005; Fowler et al., 2008) (**Figure 1C**). The parasympathetic system is activated alone during voiding, and acts to excite and contract the detrusor muscle (de Groat, 1990, 1993; Elbadawi, 1996; Holstege, 2005; Fowler et al., 2008) (**Figure 1C**). The urethral sphincter is relaxed during voiding due to the release of sympathetic activation.

The external urethral sphincter muscle is made up of striated muscle and is controlled by the somatic nervous system. The somatic motor neurons that innervate this structure have cell bodies located in the ventrolateral part of the ventral horn of the upper sacral spinal cord (S2–S4) in a nucleus called Onuf’s nucleus or the dorsolateral nucleus, depending on species (de Groat, 1990, 1993; Elbadawi, 1996; Holstege, 2005; Fowler et al., 2008). These motor neurons reach the external urethral sphincter (i.e., urethral rhabdosphincter) via the pudendal nerve.

The PMC, also called Barrington’s nucleus, is located in the dorsolateral pontine tegmentum of the brainstem and controls the function of the urinary bladder as well as other pelvic organs (Rouzade-Dominguez et al., 2003). Neurons in the PMC send direct excitatory projections to preganglionic neurons in the sacral spinal cord (de Groat, 1990, 1993; Elbadawi, 1996; Holstege and Mouton, 2003; Holstege, 2005; Fowler et al., 2008; Beckel and Holstege, 2011; Hou et al., 2016). Stimulation of the PMC leads to contraction of the detrusor smooth muscle and micturition (Hou et al., 2016). The PMC not only augments parasympathetic outflow but also attenuates output of preganglionic sympathetic and motor neurons in Onuf’s nucleus resulting in bladder contraction and sphincter relaxation (de Groat, 1990, 1993; Elbadawi, 1996; Holstege and Mouton, 2003; Holstege, 2005; Fowler et al., 2008; Beckel and Holstege, 2011). Afferent projections carrying information about bladder filling is first processed in the PAGmatter in the brainstem before being relayed to the PMC (de Groat, 1990, 1993; Elbadawi, 1996; Holstege and Mouton, 2003; Holstege, 2005; Fowler et al., 2008; Beckel and Holstege, 2011).

The micturition reflex switches the bladder from the filling phase to the emptying phase. During the filling phase, the sympathetic nervous system inhibits the detrusor smooth muscle, allowing the bladder to increase in size. In addition, the urethral sphincter contracts under background stimulation by the sympathetic nervous system maintaining continence (Holstege, 2005; Fowler et al., 2008; Beckel and Holstege, 2011). When the detrusor is relaxed, the bladder base is flat, and the urethra is pulled upward, constricting its orifice and further impeding the flow of urine. The switch to the emptying phase is triggered by tension in the urinary bladder during filling that stimulates stretch receptors (slowly adapting mechanoreceptors) within the bladder wall (Holstege, 2005; Fowler et al., 2008). These receptors activate Aδ- and C-fibers that convey sensory information about bladder distention and noxious stimuli, respectively, from the bladder neck and urethra to the lumbosacral spinal cord (L4-S4) via the pelvic, pudendal and hypogastric nerves (Holstege and Mouton, 2003; Holstege, 2005; Fowler et al., 2008; Beckel and Holstege, 2011). Aδ- and C-fibers enter the spinal cord through Lissauer’s tract and synapse in spinal cord laminae (i.e., I, V, VII, X) that contain preganglionic parasympathetic neurons as well as projection neurons that send axons to the PAG (Holstege and Mouton, 2003; Holstege, 2005; Fowler et al., 2008; Beckel and Holstege, 2011). Signals then travel from the PAG to the PMC and back to the lumbosacral spinal cord to synapse on preganglionic sympathetic and parasympathetic neurons (Holstege and Mouton, 2003; Holstege, 2005; Fowler et al., 2008; Beckel and Holstege, 2011). The PMC inhibits the preganglionic sympathetic control that releases the contraction of the sphincter and withdraws the inhibition of the detrusor. A few seconds later, there is an increase in the activity of the parasympathetic system, contracting the detrusor smooth muscle. The micturition reflex is a spinobulbospinal reflex because information travels from the spinal cord to the brainstem and back to the spinal cord to convey information regarding bladder filling (Holstege and Mouton, 2003; Holstege, 2005; Fowler et al., 2008; Beckel and Holstege, 2011).

The micturition reflex occurs without conscious control only in infants. In adults, voluntary control via higher brain centers can override the micturition reflex. This is necessary in humans for social purposes, and in animals for survival mechanisms, such as the marking of territory (de Groat et al., 1998; de Groat and Araki, 1999). Therefore, CNS control of the micturition reflex from supraspinal levels is necessary. As mentioned previously, integration occurs between the descending and ascending signals of the micturition reflex in the PAG. Several higher brain centers project to the PAG, including the hypothalamus, preoptic region, central nucleus of the amygdala, BNST, and prefrontal cortex (Beckel and Holstege, 2011). During filling, these higher brain centers can prevent the PAG from exciting neurons in the PMC and prevent voiding or incontinence (Holstege and Mouton, 2003; Holstege, 2005; Fowler et al., 2008; Beckel and Holstege, 2011).

In addition to efferent functions, the pelvic nerve contains two types of afferent fibers: C-fibers and Aδ-fibers. Aδ-fibers are myelinated fibers that transmit information to the brain about the degree of bladder distension and are essential for the generation of storage and elimination reflexes (Janig and Morrison, 1986). In contrast to the reflex underlying the storage phase, the elimination reflex relies on supraspinal circuitry as demonstrated by voiding dysfunction following suprasacral SCI (Gonzalez et al., 2014a) (**Figure 1B**). C-fibers are unmyelinated fibers that are insensitive to bladder filling under normal conditions (Gonzalez et al., 2014a). In humans, C-fibers only respond when a noxious stimulus, such as chemical irritation, is present (Fowler et al., 2008). The C-fiber reflex pathway is usually weak or undetectable in animals with an intact nervous system. Stimulation of the C-fiber bladder afferents by installation of ice water into the bladder (cold stimulation) activates voiding responses in patients with SCI (Fowler et al., 2008) (**Figure 1B**). Subcutaneous capsaicin administration blocks the C-fiber reflex in cats with SCI but does not block micturition reflexes in spinal intact cats (Fowler et al., 2008) (**Figure 1B**). Intravesical capsaicin also suppresses detrusor hyperreflexia and cold-evoked reflexes in patients with neurogenic bladder dysfunction (Fowler et al., 2008) (**Figure 1B**). In cats, the recovery of bladder function after a suprasacral SCI is mediated by a change in the afferent limb of the micturition reflex pathway and synaptic plasticity in the spinal cord (de Groat et al., 1981; Fowler et al., 2008; de Groat and Yoshimura, 2010). Following chronic SCI in cats, unmyelinated C-fiber afferents, rather than Aδ afferents, initiate voiding and a spinal micturition reflex (de Groat and Yoshimura, 2010) (**Figure 1B**). The situation in rats following SCI is more complex. In spinal intact rats and those with chronic SCI, Aδ afferents initiate the micturition reflex (Mallory et al., 1989). Although it has been demonstrated that C-fibers are not necessary for spinal micturition reflexes after SCI in rats, C-fibers do contribute to the appearance of non-voiding contractions and detrusor sphincter dyssynergia in deeply anesthetized rats with SCI (Cheng and de Groat, 2004).

Bladder afferent fibers in the pelvic nerve in rodents travel through the dorsal roots into the lateral dorsal root entry zone (i.e., Lissauer’s tract) and then give off axon collaterals that extend ventromedially and ventrolaterally along the superficial laminae of the DH. Bladder afferent fibers project to the dorsal commissure and to the SPN (laminae V–VII) that contains preganglionic parasympathetic neurons (Donovan et al., 1983; Noto et al., 1991; Steers et al., 1991; de Groat, 1993; Nadelhaft and Vera, 1995; Marson, 1997; Arms and Vizzard, 2011; Beckel and Holstege, 2011; de Groat and Yoshimura, 2012; de Groat and Wickens, 2013; Merrill et al., 2016). The ventrolateral pathway that projects along the lateral edge of the DH is referred to as the LCP of Lissauer’s tract (Morgan et al., 1981; Morgan, 1990). The medial collateral pathway, composed of afferent projections from the pudendal nerve and genital structures, projects into the dorsal commissure region (Morgan et al., 1981; Kawatani et al., 1990; Morgan, 1990).

Many bladder afferent fibers project to the SPN, synapsing with preganglionic parasympathetic neurons (Morgan et al., 1981; Morgan, 1990; Fowler et al., 2008), as well as to the dorsal commissure and superficial DH (Morgan et al., 1981; Fowler et al., 2008). The lumbosacral dorsal commissure, superficial DH, and SPN all contain interneurons important to urinary bladder function (de Groat, 1993; Fowler et al., 2008) that contribute to local circuit function as well as to supraspinal circuitry (Fowler et al., 2008). Some bladder afferents synapse with ascending pathways in the spinal cord projecting to supraspinal regions including the PAG and PMC (de Groat, 1993; Fowler et al., 2008).

There continues to be tremendous interest in growth factors (e.g., NGF, BDNF) and associated receptors (e.g., TrkA, TrkB, p75^NTR^) and the roles they play the regulation of micturition in health and disease as well as their biomarker potential for LUT disease (Dmitrieva and McMahon, 1996; McMahon, 1996; Clemow et al., 1998; Mendell et al., 1999; Vizzard, 2000b; Bjorling et al., 2001; Huang and Reichardt, 2001; Pezet and McMahon, 2006; Girard et al., 2011; Jiang et al., 2013, 2014). NGF is a potent neurotrophin that exerts pleiotropic effects in the PNS and CNS through the tissue-specific expression of TrkA and p75^NTR^ receptors. NGF regulates sensory and sympathetic neuronal development and maintenance and contributes to inflammation of somatic and visceral origin (Schnegelsberg et al., 2010; Frias et al., 2011; Ochodnicky et al., 2012). A large percentage of pelvic visceral afferent neurons, including bladder afferent neurons, exhibit neurotrophin receptors, including certain tyrosine kinase membrane receptors (Trk) for NGF and related substances (McMahon et al., 1994; Wright and Snider, 1995; Qiao and Vizzard, 2002a,b, 2005). TrkA- and TrkB-IR and Trk phosphorylation in bladder afferent neurons is increased after cystitis (Qiao and Vizzard, 2002b).

Nerve growth factor has an established role in urinary bladder inflammation (Vizzard, 2000b; Schnegelsberg et al., 2010), most likely contributing to increased voiding frequency (Dmitrieva and McMahon, 1996; Clemow et al., 1998; Chuang et al., 2001; Hu et al., 2005; Guerios et al., 2006; Zvara and Vizzard, 2007; Guerios et al., 2008). Administration of NGF intravesically (Dmitrieva et al., 1997), intrathecally (Yoshimura et al., 2006), intramuscularly (Zvara and Vizzard, 2007), or via adenovirus-mediated delivery to the urinary bladder (Lamb et al., 2004) induces increased voiding frequency as well as afferent neuronal hyperexcitability in rodents. Additionally, scavenging strategies involving sequestration of NGF or its receptor TrkA, as well as administration of Trk inhibitors, reduces urinary frequency in rodent models of urinary bladder inflammation (Dmitrieva et al., 1997; Hu et al., 2005; Klinger and Vizzard, 2008). NGF is also thought to play a role in several LUT disorders such as PBS/IC (Lowe et al., 1997; Okragly et al., 1999), OAB (Kim et al., 2006; Liu and Kuo, 2009; Liu et al., 2009, 2010, 2011) and BOO (Liu and Kuo, 2008). In fact, increased levels of NGF have been detected in the urine and the urothelium of individuals with PBS/IC, OAB, and other painful bladder conditions (Lowe et al., 1997; Okragly et al., 1999; Kuo et al., 2010). Additional NGF-mediated changes may include: modulation of local inflammatory responses through recruitment of mast cells, upregulation of neuropeptide/receptor systems and ion channels and altered expression of other neurotrophins/receptor systems (Girard et al., 2010, 2011, 2012).

A humanized monoclonal antibody (i.e., tanezumab) binds to and blocks the effects of NGF and has been used in a proof of concept human study for PBS/IC (Evans et al., 2011). Tanezumab was first developed for the treatment of osteoarthritis of the hip and knee, but further clinical trials were stopped due to adverse side effects (Cruz, 2014). Treatment produced modest improvements in self-reported pain scores as well as decreases in the number of urgency and frequency episodes. However, adverse side effects were reported in some patients, including paraesthesia (i.e., tingling skin), hyperesthesia (i.e., abnormal increase in sensitivity to stimuli), and migraines (Cruz, 2014).

## Neurochemistry of Micturition Pathways

Bladder afferents contain a number of neuroactive compounds including multiple neuropeptides (e.g., CGRP, SP, neurokinin A, neurokinin B, VIP, PACAP, cholecystokinin, enkephalins) (de Groat et al., 1983; Donovan et al., 1983; Keast and De Groat, 1992; Vizzard, 2000c, 2001; Arms and Vizzard, 2011; Merrill et al., 2013a). These neuropeptides are mainly expressed in small diameter, C-fiber afferents (de Groat et al., 1983; Donovan et al., 1983; Keast and De Groat, 1992; Vizzard, 2000c, 2001; Arms and Vizzard, 2011; Merrill et al., 2013a). The following sections will focus on the expression, distribution and functional plasticity of two members of the VIP/secretin/glucagon family of hormones in micturition reflex pathways: PACAP and VIP. The contributions of other peptides are described elsewhere (Arms and Vizzard, 2011).

Neuropeptides, expressed in afferent pathways to the LUT, exhibit either excitatory or inhibitory actions (Arms and Vizzard, 2011). Other, non-neural, sources of peptides in the LUT include the urothelium. For example, there is evidence of expression and functional activity by the VIP-PACAP family of peptides as well as the CRH family of peptides in the urothelium (Braas et al., 2006; LaBerge et al., 2006; Birder and Andersson, 2013; Hanna-Mitchell et al., 2014). The balance of peptides in LUT pathways can be affected by disease, neural injury and target organ inflammation. This change in balance of peptides can, conceivably, shift the LUT reflexes to a hyper- or a hypo-active reflex state (Arms and Vizzard, 2011). Functional changes in the micturition reflex that are demonstrated with urinary bladder inflammation (Vizzard, 2000a,c, 2001), PBS/IC, (Braas et al., 2006; de Groat and Yoshimura, 2009), OAB (Yoshimura et al., 2008), detrusor overactivity secondary to BOO (Andersson, 2006a,b), stress (Lutgendorf et al., 2000; Klausner et al., 2005; Robbins and Ness, 2008; Merrill et al., 2013b; Merrill and Vizzard, 2014; Mingin et al., 2014, 2015) or Parkinson’s disease (Hamill et al., 2012) may reflect a change in the balance of peptides in LUT reflex pathways.

## Pituitary Adenylate Cyclase-Activating Polypeptide (PACAP) and PAC1, VPAC1, VPAC2 Receptors

Pituitary adenylate cyclase-activating polypeptide is a member of the VIP/secretin/glucagon family of super hormones that was originally isolated from hypothalami based on its stimulation of anterior pituitary AC activity (Ogi et al., 1990; Arimura, 1998). The rat PACAP precursor protein undergoes post-translational processing to produce two, ∝-amidated forms: PACAP38 and PACAP27 (Kimura et al., 1990; Ohkubo et al., 1992; Okazaki et al., 1995; Arimura, 1998; Braas et al., 1998). The distribution of these two forms is tissue-specific with PACAP38 being the predominate form (Arimura et al., 1991; Arimura, 1998). PACAP38 is highly conserved among mammalian species underscoring its important involvement in cell signaling, modulation and trophic functions in the nervous and endocrine systems (Moller et al., 1997b; Arimura, 1998). There are three distinct G-protein-coupled receptors for PACAP and VIP: PAC1, VPAC1, and VPAC2 (Ishihara et al., 1992; Hashimoto et al., 1993; Hosoya et al., 1993; Lutz et al., 1993; Spengler et al., 1993; Inagaki et al., 1994). The expression of PAC1 and VPAC receptors is tissue- and cell type-specific (May and Braas, 1995; Braas and May, 1996, 1999; Beaudet et al., 1998, 2000; Braas et al., 1998; May et al., 1998; DiCicco-Bloom et al., 2000). After G-protein-coupled PAC1 receptor activation and signaling at the plasma membrane, the receptor complex is often rapidly internalized via endocytic vesicles for trafficking into various intracellular compartments and pathways. PACAP mediates its diverse cellular functions through internalization of its cognate G-protein-coupled PAC1 receptor and endosomal signaling (May and Parsons, 2017).

## PACAP and PAC1 Receptor Neuronal Functions in the LUT

Pituitary adenylate cyclase-activating polypeptide peptides exhibit diverse functions in many organ systems (e.g., endocrine, nervous, urinary, gastrointestinal, cardiovascular systems) and PACAP peptides are widely expressed in CNS and PNS, including sensory and autonomic ganglia (Koves et al., 1990, 1991; Arimura et al., 1991; Ghatei et al., 1993; Masuo et al., 1993; Tatsuno et al., 1994; May and Braas, 1995; Portbury et al., 1995; Shiotani et al., 1995; Braas and May, 1996, 1999; Holgert et al., 1996; Klimaschewski et al., 1996; Sundler et al., 1996; Brandenburg et al., 1997; Moller et al., 1997a,b; Nogi et al., 1997; Arimura, 1998; Beaudet et al., 1998, 2000; Braas et al., 1998; May et al., 1998; Cheppudira et al., 2009). PACAP-IR is expressed in nerve fibers within the urinary bladder smooth muscle, suburothelial nerve plexus and surrounding blood vessels (Fahrenkrug and Hannibal, 1998b). PACAP expression is significantly reduced in the urinary tract (i.e., ureter, bladder, and urethra) by neonatal capsaicin treatment delivered intraperitoneally, suggesting these fibers are derived from small diameter, C-fiber neurons (Fahrenkrug and Hannibal, 1998b). PACAP is expressed in DRG under control conditions and PACAP expression is increased after nerve injury or inflammation (Zhang et al., 1995, 1996; Larsen et al., 1997; Moller et al., 1997a; Vizzard, 2000c). Immunohistochemistry studies that demonstrated PACAP expression in LUT pathways and increased expression after SCI or bladder inflammation used both monoclonal and polyclonal PACAP antisera. Specificity of these reagents was confirmed with antibody preabsorption with PACAP (20 mg/ml) peptide (Hannibal et al., 1995; Calupca et al., 2000; Vizzard, 2000c) and in PACAP^-/-^ mice (Girard et al., 2006). Using behavioral and neurological tests, we have demonstrated that PACAP^-/-^ and VIP^-/-^ mice exhibit functional distinctions between the knockout genotypes. These results suggest that PACAP and VIP have evolved to possess distinct biological activities with the respective knockout phenotypes representing deficits unmitigated by the actions of the complementary related peptide (Girard et al., 2006).

## PACAP-Mediated Effects on Urothelium

A disruption to the barrier function of the urothelium (Negrete et al., 1996; Zeidel, 1996) may occur with disorders and injury that affect the bladder including PBS/IC or spinal cord injury (Lavelle et al., 2000; Apodaca et al., 2003, 2007). This loss of barrier integrity may contribute to the altered sensory processing (i.e., allodynia) observed in cystitis (i.e., pain with low to moderate bladder distention). The urothelium expresses PAC1 receptors and with stimulation, releases ATP to stimulate receptors on underlying sensory nerve fibers in the suburothelial plexus (Girard et al., 2008b). ATP release was evoked by PACAP27, PACAP38, and VIP application to cultured urothelial cells whereas PACAP27 and PAC1 receptor antagonism blocked ATP release (Girard et al., 2008b). PACAP signaling through PAC1 receptors may regulate micturition reflex function at the level of the urothelium (Girard et al., 2008b).

## PACAP or VIP Knockout (^-/-^) Mice Exhibit Altered Micturition Reflexes

Mice with a genetic disruption or deletion of PACAP or VIP exhibit altered bladder and somatic function. PACAP^+/-^ and PACAP^-/-^ mice display reduced mechanical sensitivity in the pelvic and hindpaw regions as determined with von Frey monofilament testing (May and Vizzard, 2010). These differences may reflect distinct roles for VIP and PACAP in bladder sensory function and referred somatic sensation in the mouse. In contrast to somatic mechanosensitivity differences, both PACAP^-/-^ and VIP^-/-^ mice exhibited urinary bladder hypertrophy (Jensen et al., 2008; May and Vizzard, 2010). Cystometric analyses in PACAP^-/-^ and VIP^-/-^ mice demonstrated increased bladder capacity, void volume, and longer duration between micturition events (i.e., intercontraction interval) (May and Vizzard, 2010) (Studeny et al., 2008). In addition to urinary bladder hypertrophy and increased bladder mass, VIP^-/-^ mice have increased permeability to urea, increased basal expression of NGF and an exaggerated pro-inflammatory response (Girard et al., 2008a; Jensen et al., 2008; Studeny et al., 2008). Additionally, studies suggest an increase in bladder afferent activity in lumbosacral DRG in VIP^-/-^ mice demonstrated with a marker of cellular activation (i.e., phosphorylated cAMP response-element binding protein) (Jensen et al., 2008). Given that PACAP^-/-^ and VIP^-/-^ mice are global knockout models, the contributions of these peptides to LUT function from CNS or PNS sites of action must both be considered.

Urinary bladder dysfunction presents a major problem in the clinical management of patients suffering from a large number of neurological injuries (e.g., upper motor neuron disease after spinal cord injury, stroke), disorders (e.g., multiple sclerosis, Parkinson’s disease) and chronic pain syndromes (e.g., PBS/IC). Information related to the normal organization of the micturition reflex and neuroplasticity with injury, disease and/or inflammation has the tremendous potential to increase our understanding of bladder disorders, to identify novel targets and to develop new therapeutic approaches.

## Cyclophosphamide (CYP)-Induced Cystitis

**FIGURE 2:**
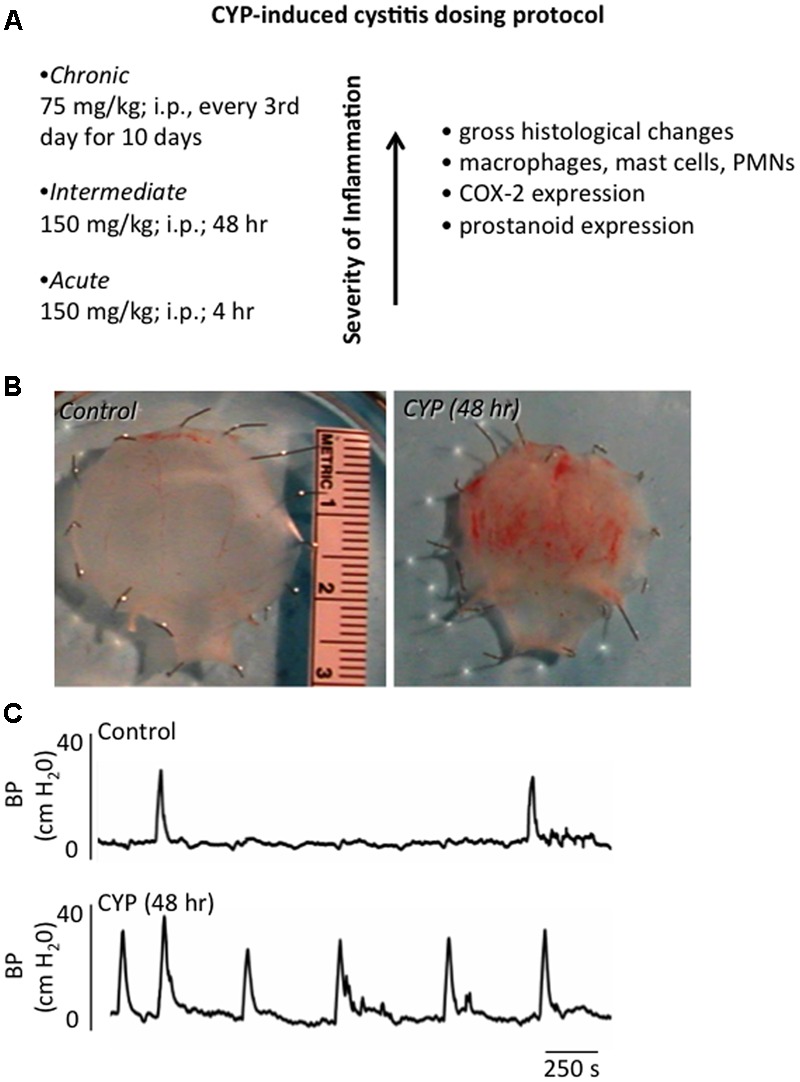
Inflammation of the Urinary Bladder Induced by CYP Treatment. **(A)** Chemical cystitis is induced in rats by CYP, which is metabolized to acrolein, an irritant eliminated in the urine. CYP is administered intraperitoneally in one of three dosing protocols to elicit: acute (4 h), intermediate (48 h) or chronic (every 3rd day for 10 days) inflammation. **(B)** Gross microscopic analyses of urinary bladders from animals treated with CYP revealed: mucosal erosion, ulcerations, edema, and, in some instances, petechial hemorrhages. Histological changes observed after chronic CYP treatment included the presence of inflammatory cell infiltrates, including mast cells, macrophages and neutrophils. **(C)** CYP-induced cystitis increased voiding frequency in rodents. Representative cystometrogram recordings using continuous, intravesical infusion of saline in conscious rats with an open outlet from a control rat and a rat treated with CYP (48 h) are shown. Bladder pressure in a control (top) and CYP-treated (bottom) rat are shown. Figure is based upon the CYP-induced cystitis phenotype (Vizzard, 2000a; Braas et al., 2006; Klinger et al., 2007).

The etiology of PBS/IC is unknown. Thus, there is no universally accepted animal model for PBS/IC and no one model completely mimics all signs and symptoms of PBS/IC (Westropp and Buffington, 2002). CYP-induced cystitis is one of the most widely used animal models to study various aspects of the human condition, PBS/IC (**Figure 2**). CYP is an urotoxic anti-tumor agent that requires metabolism in the liver by cytochrome P50 (Boucher et al., 2000; Batista et al., 2006; Wantuch et al., 2007) and is then metabolized into acrolein, the urinary metabolite (Boucher et al., 2000; Eichel et al., 2001; Batista et al., 2006). It has been proposed that the urothelial damage that results from CYP treatment occurs by direct contact of the urothelium with acrolein (**Figure 2B**). This contact produces toxic effects on the bladder wall including edema, ulceration, neovascularization, necrosis, and hemorrhagic cystitis that are characteristic of treatment with CYP (Boucher et al., 2000; Eichel et al., 2001; Batista et al., 2006). CYP treatment has also been shown to produce changes in histological, permeability, and functional aspects of the urinary bladder (Boucher et al., 2000; Eichel et al., 2001; Batista et al., 2006). Functional changes after CYP administration include irritative voiding patterns in humans and animals, as well as an increase in micturition frequency in rodents (Cox, 1979; Maggi et al., 1992; Vizzard, 2000a,b, 2001; Eichel et al., 2001; Qiao and Vizzard, 2002b; Murray et al., 2004; Hu et al., 2005; Braas et al., 2006) (**Figure 2C**). CYP-induced cystitis is thought to alter bladder reflexes through stimulation of capsaicin-sensitive bladder afferent nerves (Yamamoto et al., 2012). CYP does not produce histological lesions or signs of inflammation in other tissues apart from the bladder, but does produce visceral pain similar to PBS/IC (Boucher et al., 2000; Wantuch et al., 2007). Our laboratory has found changes in urinary bladder function, somatic sensation, and the inflammatory milieu of the urinary bladder with various CYP treatment protocols in rodents (i.e., rats and mice) (**Figure 2A**). However, differences exist between the model and PBS/IC, including severity of inflammation at the level of the urinary bladder (Yamamoto et al., 2012). Although the naturally occurring FIC also models the human condition of PBS/IC (Westropp and Buffington, 2002), FIC cats are not available to most investigators and FIC cats usually represent established disease. Thus, determining early, initiating events and mechanisms involved in the disease process is difficult. The CYP-induced bladder inflammation model enables the testing of specific hypotheses related to LUT function.

## Neuroplasticity of PACAP/Receptor Expression and Function with Cystitis

It has been previously demonstrated that PACAP and its receptors are regulated by CYP-induced cystitis and may contribute to the development of urinary bladder dysfunction (Braas et al., 2006). Following a down regulation in transcript expression after acute (4 h) CYP-induced cystitis, PACAP and PAC1 transcript expression are dramatically upregulated in the urothelium, L6 and S1 DRG and detrusor smooth muscle after intermediate (48 h) or chronic (10 days) CYP-induced cystitis (Girard et al., 2008b). In contrast, VPAC1 and VPAC2 transcript expression remains upregulated in the urothelium and detrusor smooth muscle with acute and intermediate CYP-induced cystitis but down-regulation of VPAC2 transcript expression occurs with chronic treatment (Girard et al., 2008b). PACAP-IR in the spinal cord is increased in lumbosacral spinal cord regions [e.g., superficial laminae (I–II) of the DH, LCP of Lissauer, SPN] associated with LUT reflexes following CYP-induced cystitis (Vizzard, 2000c; Herrera et al., 2006). Additionally, numbers of bladder afferent cells exhibiting PACAP-IR increased in lumbosacral DRG following CYP-induced cystitis (Vizzard, 2000c).

Pharmacological studies using PAC1 receptor antagonists suggest that PACAP and its receptors may play a role in bladder dysfunction with bladder inflammation (Braas et al., 2006). Intrathecal (L6-S1) or intravesical administration of a PAC1 receptor antagonist, PACAP(6-38), increased bladder capacity but not intravesical pressure with CYP-induced cystitis (Braas et al., 2006). The different routes of administration (i.e., intrathecal and intravesical) with similar functional improvements suggest that PACAP(6-38) may be acting at multiple sites in the PNS and CNS. Intrathecal administration of PACAP(6-38) may be acting on superficial DH neurons to block PACAP release from C-fiber afferents, whereas, intravesical PACAP(6-38) may be targeting urothelial cells, suburothelial nerve fibers or detrusor smooth muscle cells (Braas et al., 2006). Intrathecal or intravesical blockade of PACAP/PAC1 may be a promising target to reduce voiding frequency with cystitis.

## PACAP Expression in LUT with CYP-Induced Cystitis in PACAP Promoter-Dependent EGFP BAC Transgenic Mice

The PACAP-EGFP mouse strain is a transgenic line in which PACAP expressing cells and neurons are tagged with green fluorescent protein for easy visualization and tracking (Condro et al., 2016). PACAP-EGFP transgenic mice, Tg(Adcyap1-EGFP)FB22Gsat/Mmucd (**RRID**:IMSR_MMRRC:012011) were generated using the BAC (RP24-358O1) by the Gene Expression Nervous System Atlas (GENSAT) project and obtained from the Mutant Mouse Resource and Research Centers (Condro et al., 2016). The identification of PACAP expressing neurons has been challenging using standard immunocytochemistry and *in situ* hybridization techniques especially in areas with low peptide expression levels. The availability of these mice obviates these difficulties and will allow ready identification of these neurons and their fiber tracts to establish neurocircuits. The PACAP-EGFP construct is regulated by the endogenous promoter. Therefore, it is possible to examine whether altered physiology, including stress, can differentially regulate specific neuronal PACAP populations in the CNS and PNS. We previously demonstrated an upregulation of PACAP expression in rodent micturition pathways following CYP-induced cystitis (Braas et al., 2006). We have subsequently examined the effects of CYP-induced cystitis (4 h, 48 h, chronic) in PACAP promoter-dependent EGFP BAC transgenic mice (gift from Dr. James A. Waschek, David Geffen School of Medicine, University of California, Los Angeles) (May et al., 2015, 2017a; Gonzalez et al., 2016). In control mice with no CYP treatment, low basal expression of PACAP-EGFP+ fibers and cells was present in the superficial DH (L1, L2, L4-S1) and DRG (L1, L2, L6, S1) examined. After CYP-induced cystitis, numbers of PACAP-EGFP+ cells increased dramatically in spinal cord segments and DRG (L1, L2, L6, and S1) involved in micturition reflexes. PACAP-EGFP+ nerve fibers were increased in density in the superficial laminae (I-II) of the DH and LCP of the lumbosacral spinal cord. Following CYP-induced cystitis, numbers of PACAP-EGFP+ urothelial cells increased with the duration of cystitis (May et al., 2015, 2017a). After CYP-induced cystitis, additional changes in PACAP-EGFP+ nerves fibers and cells were observed in numerous supraspinal locations including: locus coeruleus, Barrington’s nucleus, rostral ventrolateral medulla, PAG, raphe, and amygdala (May et al., 2015, 2017a). PACAP expression, in central and peripheral LUT pathways, may play a role in altered visceral sensation (allodynia) and/or increased voiding frequency in CYP-induced cystitis and in the chronic inflammatory pain syndrome, PBS/IC.

## Role of Nerve Growth Factor (NGF) and Associated Receptors in LUT Plasticity

Nerve growth factor is upregulated at the site of tissue injury, inflammation and/or target organ hypertrophy (Heumann et al., 1987; Lewin and Mendell, 1993; Meller and Gebhart, 1994; Dray, 1995; Woolf et al., 1997) and is also released from the target organ for TrkA binding and retrograde transport in DRG afferent neurons (Johnson et al., 1987). This increase in NGF expression or increased uptake of NGF in the DRG neurons may then induce increased production of neuropeptides (e.g., SP, CGRP, and PACAP) and alter sensory transduction (Donnerer et al., 1992; Woolf et al., 1997). A large percentage of pelvic visceral afferent neurons, including bladder afferent neurons, express neurotrophic factor receptors, including Trk and p75^NTR^ for NGF and proNGF binding (McMahon et al., 1994; Wright and Snider, 1995; Qiao and Vizzard, 2002a,b). Similar to increased expression of NGF after cystitis, increased expression of TrkA- and TrkB-IR and Trk phosphorylation in bladder afferent neurons has been demonstrated (Qiao and Vizzard, 2002b).

## NGF and PACAP Interactions

There is a growing body of literature that supports reciprocal regulatory interactions between NGF and PACAP in pheochromocytoma (PC)12 cells and sensory ganglia. NGF is a positive regulator of PACAP expression in DRG cells (Jongsma Wallin et al., 2001). In rat PC12 cells, both NGF and PACAP can induce PC differentiation into a neuronal phenotype (Grumolato et al., 2003). Similarly, following transfection of PC12 cells with a PACAP promoter-luciferase construct, exogenously applied PACAP and/or NGF upregulated PACAP gene expression (Hashimoto et al., 1993; Yamamoto et al., 2012). In addition, the neurotrophins, NGF and/or BDNF can also facilitate expression of the PACAP-selective PAC1 receptor in CNS neurons (i.e., cerebellar granule cells) and PC12 cells (Jamen et al., 2002). In complementary studies, PACAP upregulated neurotrophin receptors, TrkA and TrkB, expression and/or phosphorylation in PC12 cells and CNS neurons (i.e., hippocampal neurons) in a Src-dependent manner (Lee et al., 2002). Using sympathetic neuroblasts, PACAP was shown to augment TrkA and TrkC expression in neuronal differentiation (DiCicco-Bloom et al., 2000). Reciprocal regulation of PACAP and NGF signaling pathways may be a feed-forward mechanism to amplify critical, physiological processes (e.g., survival or differentiation) during neuronal development or regeneration. In the context of urinary bladder inflammation, the same feed-forward mechanism may be detrimental resulting in the amplification of painful signals and exacerbation of target organ dysfunction.

## Transgenic Mouse Model of Chronic, Urothelial NGF Overexpression (NGF-OE)

**FIGURE 3:**
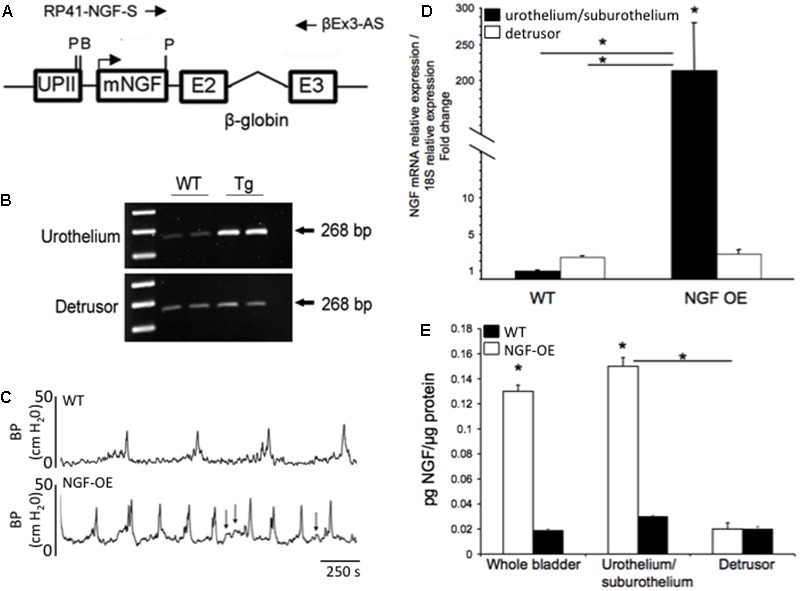
Generation of NGF-overexpressing (NGF-OE) transgenic mice. **(A)** A 6,058 bp NotI fragment containing the uroplakin II (UPII)-NGFv2 transgene was microinjected into the pronuclei of fertilized C57BL/6J embryos to generate NGF-OE transgenic F0 lines. BamHI and Pst I restriction sites, and the position of the primers used for genotyping (RP41-NGF-S and βEx3-AS) are indicated (Schnegelsberg et al., 2010). **(B)** Representative semi-quantitative RT-PCR data showing amplification of total NGF mRNA (268 bp) isolated from the urothelium or detrusor smooth muscle bladder of WT and NGF-OE mice (Schnegelsberg et al., 2010). **(C)** Open cystometry in conscious, unrestrained WT and NGF-OE mice demonstrated increased voiding frequency and non-voiding contractions (NVCs) in NGF-OE mice (Schnegelsberg et al., 2010). Representative cystometrogram traces from WT (top) and NGF-OE (bottom) mice during continuous intravesical infusion of room temperature saline. Bladder pressure (BP, cm H_2_O) is shown. Arrows indicate examples of NVCs. **(D,E)** Characterization of NGF mRNA and protein expression in urinary bladder from NGF-OE mice by quantitative RT-PCR and ELISAs (Cheppudira et al., 2008). **(D)** NGF transcript expression in urothelium/suburothelium and detrusor layers in NGF-OE and WT mice normalized to the housekeeping gene, 18S. Values are means ± SEM. ^∗^*P* ≤ 0.001: NGF-OE urothelium/suburothelium vs. WT urothelium/suburothelium and detrusor (with horizontal bars) and within NGF-OE bladder layers (without horizontal bars) (Cheppudira et al., 2008). **(E)** Summary histogram of NGF protein expression in whole urinary bladder, urothelium/suburothelium, and detrusor layers in NGF-OE and WT mice. Values are means ± SEM. ^∗^*P* ≤ 0.001: urothelium/suburothelium vs. detrusor from NGF-OE mice (with horizontal bars) and NGF-OE vs. WT (without horizontal bars) (Cheppudira et al., 2008).

Numerous studies have pointed to NGF as a molecule of interest in urinary bladder dysfunction and specifically, PBS/IC. Studies have demonstrated: (1) significant increases in urinary bladder NGF, after acute and chronic bladder inflammation induced by CYP (Vizzard, 2000b); (2) increased voiding frequency, reduced bladder capacity and increased Fos protein in lumbosacral spinal interneurons after exogenous delivery of NGF into the detrusor smooth muscle (Zvara and Vizzard, 2007); (3) reduced voiding frequency with NGF-scavenging agents in CYP-treated rats (Hu et al., 2005) and (4) increased expression of NGF in urine of women and in the urothelium of bladder biopsies from women with PBS/IC (Lowe et al., 1997; Okragly et al., 1999). To more closely mimic the environment of chronic bladder inflammation and the clinical syndrome of PBS/IC, mice with chronic overexpression of NGF in the urothelium (uroplakin II NGF transgenic mice) were generated at Roche Palo Alto under the direction of Dr. Debra Cockayne and in collaboration with Dr. Henry Sun at New York University Medical School (**Figures 3A,B**) (Schnegelsberg et al., 2010). Mice with chronic, urothelial NGF-OE are valuable tools in determining the contribution of increased NGF expression in the urothelium to neural and functional plasticity of micturition reflexes (**Figures 3D,E**). Functionally, NGF-OE mice exhibit frequent urination and the presence of non-voiding bladder contractions as well as referred somatic pelvic hypersensitivity (Schnegelsberg et al., 2010) (**Figure 3C**). The electrical properties of the MPG neurons were unchanged suggesting that the efferent limb of the micturition reflex does not influence the voiding function in NGF-OE mice (Girard et al., 2012). Pleiotropic changes, subsequent to NGF-OE, including changes in the expression of growth factors, neuroactive compounds, receptors and ion channels (e.g., TRP channels) (Allen and Dawbarn, 2006; Pezet and McMahon, 2006) can also directly modulate pain and bladder/visceral sensory function and could contribute to altered urinary bladder function in NGF-OE mice (Yoshimura et al., 2002; Ford et al., 2006; Pezet and McMahon, 2006). For example, PACAP/VIP and receptor expression is changed in micturition pathways in NGF-OE mice (Arms et al., 2010). The PAC1 mRNA and PAC1-IR were upregulated whereas PACAP mRNA and PACAP-IR were decreased in urothelium of NGF-OE mice (Arms et al., 2010). In contrast, VPAC1 mRNA was decreased in both urothelium and detrusor smooth muscle of NGF-OE mice (Arms et al., 2010). VIP mRNA expression and VIP-IR were not altered in urinary bladder of NGF-OE mice (Arms et al., 2010). These additional changes in PACAP and associated receptors in micturition pathways of NGF-OE mice may also contribute to altered urinary bladder function in NGF-OE.

## Contributions of PACAP/Receptor Signaling to Increased Voiding Frequency and Somatic Sensitivity in NGF-OE

Chronic, urothelial NGF-OE, achieved through the use of a highly urothelium-specific uroplakin II promoter, produces neuroanatomical and functional changes in the LUT (**Figure 3**). Chronic, urothelial NGF-OE results in: (1) hyperinnervation in the urinary bladder; (2) increased voiding frequency; (3) increased presence and amplitude of non-voiding contractions during the filling phase and (4) increased referred somatic sensitivity (Schnegelsberg et al., 2010) (**Figure 3**). Previous sections have described the following key observations concerning PACAP/receptor signaling in the LUT: (1) PAC1-immunoreactive fibers and neurons are present in the LUT including the urinary bladder and DRG (Braas et al., 2006); (2) PAC1 receptor is expressed by the DRG, urothelium, and detrusor smooth muscle (Braas et al., 2006); (3) PACAP increases detrusor contractions (Braas et al., 2006) and (4) PAC1 receptor antagonists, delivered intrathecally or intravesically, reduce voiding frequency in rodents with CYP-induced cystitis (Braas et al., 2006). Thus, we evaluated whether PACAP/receptor signaling contributes to increased voiding frequency and somatic sensitivity in NGF-OE mice (Girard et al., 2016). Intravesical administration of the PAC1 receptor antagonist, PACAP(6-38) (300 nM), increased intercontraction interval (2.0-fold) and void volume (2.5-fold) and reduced pelvic sensitivity in NGF-OE mice (Girard et al., 2016) but had no effects in WT mice. PACAP/receptor signaling contributes to the increased voiding frequency and pelvic sensitivity observed in NGF-OE mice.

We have also determined whether additional changes in neuropeptides/receptors and growth factors/receptors are observed in LUT pathways in NGF-OE treated *with* CYP to induce cystitis (Girard et al., 2012). Quantitative PCR was used to determine NGF, BDNF and receptors (TrkA, TrkB, p75^NTR^) and PACAP/VIP and receptors (PAC1, VPAC1, VPAC2) transcripts expression in LUT tissues from NGF-OE and WT mice with CYP-induced cystitis (4 h, 48 h, and chronic) (Girard et al., 2012). As expected, NGF mRNA in the urothelium was increased in control NGF-OE mice. However, urothelial expression of NGF mRNA in NGF-OE mice treated with CYP was not further increased but maintained with all durations of CYP (Girard et al., 2012). In contrast, CYP-induced cystitis in NGF-OE mice resulted in significant, additional changes in transcript expression for NGF, BDNF and receptors (TrkA, TrkB, p75^NTR^) and PACAP/VIP and receptors (PAC1, VPAC1, VPAC2) in lumbosacral DRG and urinary bladder (e.g., urothelium, detrusor) that was dependent on the duration of cystitis (Girard et al., 2012). Conscious cystometry in unrestrained, NGF-OE mice treated with CYP demonstrated significant increases in voiding frequency above that observed in control NGF-OE mice underscoring that bladder functional changes were not saturated (Girard et al., 2011). These results suggest that chemical mediators (e.g., neurotrophins, neuropeptides) upregulated when CYP-induced cystitis is combined with NGF-OE can contribute to neurochemical and functional LUT plasticity in NGF-OE mice (Guerios et al., 2006, 2008; Arms et al., 2010, 2013; Schnegelsberg et al., 2010; Arms and Vizzard, 2011; Gonzalez et al., 2013, 2014a,b).

## PACAP Mechanisms and Stress Facilitate Micturition Dysregulation

Patients with disorders of the LUT and associated disease states report worse symptoms during stress (Westropp and Buffington, 2002; Nazif et al., 2007). Several studies support this role in the exacerbation and likely the development of a number of LUT disorders including OAB syndrome and PBS/IC (Birder and Andersson, 2013). A majority of these patients report exacerbation of symptoms by clinical stress (Rothrock et al., 2001a,b), and experimental stress increases bladder pain and urgency in these individuals (Lutgendorf et al., 2000). In addition, animal models of stress demonstrate symptoms of bladder dysfunction (e.g., increased micturition frequency, urgency, pain) as well as anxiety-like behavior (Birder and Andersson, 2013) that may be due, in part, to disruption of the HPA axis. Cortisol, by feeding back on the HPA axis, normally acts to attenuate inflammation; however, abnormalities in the feedback loop may cause dysregulation of the inflammatory response. Therefore, patients with bladder dysfunction disorders may have abnormalities in the HPA axis, and stress could be attributed to the increase in bladder symptoms, like urgency and pain, reported by these patients (Westropp and Buffington, 2002; Nazif et al., 2007). However, while stress has been associated with symptom aggravation, the pathophysiology underlying the effect of stress on urinary frequency and/or other voiding disorders remains unknown.

## Stress Effects on the Structure and Function of the Urinary Bladder

Stress is known to exacerbate symptoms of PBS/IC, and exposure to stressors is related to the onset, progression and even outcome of many disease states including those related to the urinary bladder (Yamamoto et al., 2012). Several animal models of stress, including social stress (Miczek, 1979; Bhatnagar et al., 2006; Chang et al., 2009; Wood et al., 2009), immobilization stress (Spanos et al., 1997; Alexacos et al., 1999; Boucher et al., 2002, 2010), WAS (Cetinel et al., 2005; Robbins et al., 2007; Smith et al., 2011), and electric footshock (Imaki et al., 1991; Robbins and Ness, 2008; Black et al., 2009) produce morphological changes in the urinary bladder, urinary bladder dysfunction, urothelial barrier disruption, inflammation, and visceral sensitivity similar to signs and symptoms of PBS/IC.

## Psychosocial Stress, Altered Behaviors and Physiological Disorders

From the different modes of stress, psychosocial stress is the most familiar and relevant to everyday challenges and experiences. Unlike physical and metabolic stress which reflects perturbations of the homeostatic or resting state of the body, psychosocial stress results from our cognitive appraisal of some perceived threat (either real or imagined) and our judgment that we may not have the resources or means to overcome that challenge (Marshall and Garakani, 2002; Turner-Cobb, 2005; FitzGerald et al., 2009; Lovallo, 2013; Mahon et al., 2013; Mihaljevic et al., 2016; Stephens et al., 2016). Classically, the heightened HPA axis coordinates sympathetic nervous system and cortisol adaptive responses to maintain homeostasis (allostasis) (Marshall and Garakani, 2002; Turner-Cobb, 2005; FitzGerald et al., 2009; Lovallo, 2013; Mahon et al., 2013; Mihaljevic et al., 2016; Stephens et al., 2016); however, repeated challenges or the inability to attenuate stress signaling even when the dangers have dissipated can result in maladaptations and cumulative long-term damages (i.e., increased HPA and sympathetic reactivity; allostatic overload) that can manifest a variety of disorders (Marshall and Garakani, 2002; Turner-Cobb, 2005; FitzGerald et al., 2009; Lovallo, 2013; Mahon et al., 2013; Mihaljevic et al., 2016; Stephens et al., 2016). Psychosocial stressors engage the same pathways, and as in other stress modalities, the culmination of the many sustained psychosocial insults on individuals can increase the risk of a panoply of behavioral and physiological disorders including anxiety, depression, altered feeding preferences and behaviors, cardiovascular disease, obesity/metabolic diseases, gastrointestinal/urinary dysregulation, immunologic disorders and tumor progression (Herman et al., 1996, 2003, 2005, 2012, 2016, Marshall and Garakani, 2002; Turner-Cobb, 2005; Ressler and Mayberg, 2007; Jankord and Herman, 2008; FitzGerald et al., 2009; Jeanneteau et al., 2012; Lovallo, 2013; Mahon et al., 2013; Mihaljevic et al., 2016; Stephens et al., 2016). Juveniles and adolescents are particularly vulnerable to stress- and trauma-related effects on neural development; therefore, psychosocial stress during these life stages may have long-lasting consequences with enhanced risks for future behavioral and physiological disorders (Ressler and Mayberg, 2007). Thus, psychosocial stress has a broad impact on health and disease progression with substantial human and societal costs (Herman et al., 1996, 2003, 2005, 2012, 2016; Jankord and Herman, 2008; Jeanneteau et al., 2012).

Yet despite the consequences of psychosocial stressors, some of the fundamental neural mechanisms and pathways linking psychosocial stress to altered behaviors and physiological disorders are still unclear. Although a large body of data have implicated CRH, arginine vasopressin, mineralocorticoid and glucocorticoid receptors, FKBP5 (a co-chaperone with hsp90 in GR heterocomplex), catecholamine metabolic enzymes, dopamine D2 receptor, and serotonin transporter isoforms transcripts in stress (Valentino, 1988; Marshall and Garakani, 2002; Turner-Cobb, 2005; FitzGerald et al., 2009; Lovallo, 2013; Mahon et al., 2013; Mihaljevic et al., 2016; Stephens et al., 2016), how altered expression of these genes contribute to the stress-related disorders remains to be elucidated. However, among central neuroregulators, PACAP and its cognate G protein coupled PAC1 receptor have recently been implicated as novel stress mediators. Ressler et al. (2011) published an elegant study that demonstrated the critical involvement of PACAP/PAC1 receptor expression and signaling in regulating psychological and physiological responses to traumatic stress. PACAP/PAC1 receptor transcripts are increased in specific limbic structures following RVS exposure; CNS PACAP signaling is anxiogenic and anorexic (Hammack et al., 2009; Lezak et al., 2014a,b; Missig et al., 2014; Roman et al., 2014), and chronic stress-induced anxiety-related behaviors can be blocked by PAC1 receptor antagonists. These PACAP-mediated responses are very similar to the anxiogenic effects of CRH suggesting that the two peptidergic systems may be integrated in limbic pathways. But importantly, unlike CRH, recent work demonstrated that the PACAPergic system is dysregulated in PTSD in a sex-specific manner (Ressler et al., 2011). Recent studies suggest potential interactions between stress, PACAP and circulating gonadal hormones to differentially regulate the PACAPergic system in men and women with pathology including PTSD (King et al., 2017). Blood PACAP levels correlated with severity of PTSD symptoms and a single nucleotide polymorphism in the estrogen response element of the PAC1 receptor gene is predictive of PTSD diagnosis and symptoms in females (Ressler et al., 2011). These observations are consistent with the anxiolytic behavioral phenotype of PACAP knockout mice (Hashimoto et al., 2001; Otto et al., 2001; Hattori et al., 2012) and importantly, recent PACAP knockout studies have implicated PACAP as a unique mediator of emotional stressors (Hammack et al., 2009; Lezak et al., 2014a,b; Missig et al., 2014; Roman et al., 2014). From these aggregate results, the novel identification of PACAP/PAC1 receptor in emotional stress adds an important layer to existing neural circuits and mechanisms, which may further enhance understandings of stress and disease pathways.

**FIGURE 4:**
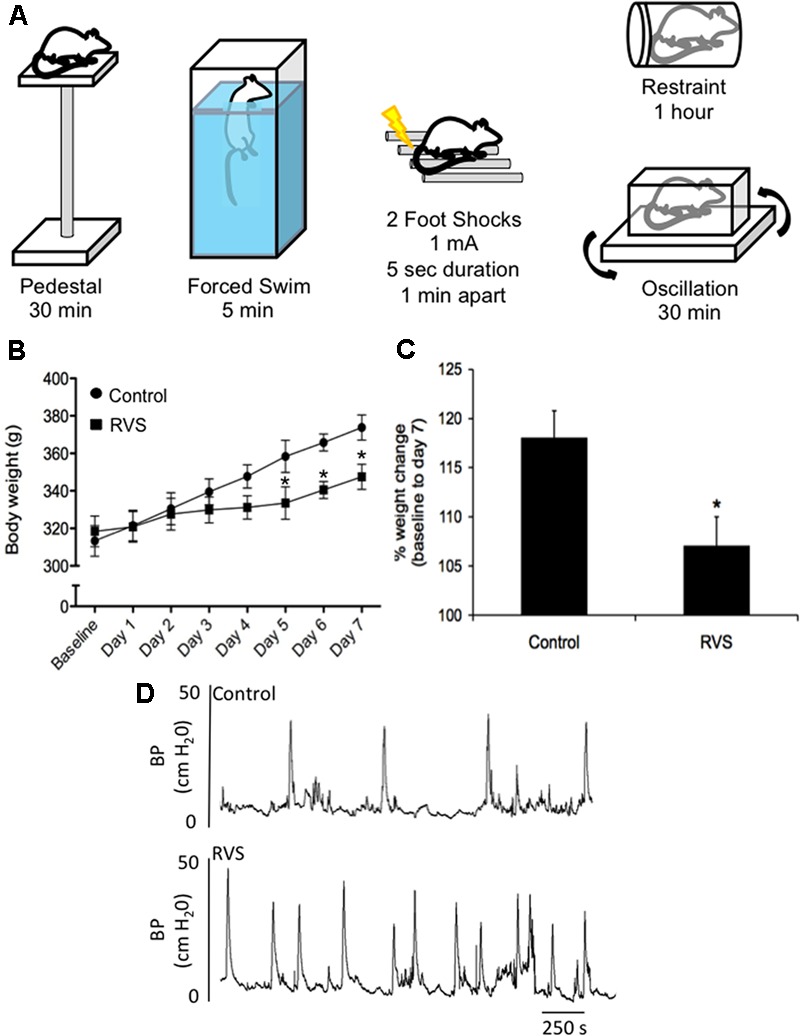
Repeated variate stress (RVS) protocol (7 days). **(A)** Five different stressors and the duration of each stressor for each day they are administered in the RVS protocol (Merrill et al., 2013b; Longden et al., 2014a,b; Merrill and Vizzard, 2014). Swim and footshock stressors are repeated on the last 2 days of RVS. s, seconds; min, minutes. **(B)** Changes in body weight of rats during 7 days of RVS. On days 5–7 of stressor exposure, rats exposed to RVS exhibit significant weight gain attenuation compared with controls rats. Body weights were significantly (*P* ≤ 0.01) decreased in the RVS group on days 5–7 of stress (Merrill et al., 2013b). **(C)** Percentage weight change from baseline body weight to body weight on day 7 of stressor exposure was significantly (*P* ≤ 0.01) decreased in rats exposed to RVS. Values are means ± SEM, ^∗^*P* ≤ 0.01 (Merrill et al., 2013b). **(D)** Representative cystometrogram recordings using continuous, intravesical infusion of saline in conscious rats with an open outlet from a control rat and a rat exposed to RVS demonstrate increased voiding frequency in rats exposed to RVS. Bladder pressure in a control (top) rat and RVS-exposed (bottom) rat are shown (Merrill et al., 2013b).

A RVS paradigm has been previously used to examine PACAP and BDNF mRNA expression in the BNST (Hammack et al., 2009; King et al., 2017) (**Figure 4A**). This model of repeated stress was found to be anxiogenic, a result most likely mediated by BNST PACAP (Hammack et al., 2009; Lezak et al., 2014a,b; Missig et al., 2014; Roman et al., 2014). This paradigm involves seven consecutive days with daily exposure to one of five different stressors: oscillation, swim, footshock, restraint, and pedestal stress (**Figure 4A**). Oscillation stress involves rodents placed inside a chamber secured to a rotator on low to medium speed for 30 min. Rodents are placed on an elevated platform for 30 min during pedestal stress. The other stressors include 5 min of forced monitored swimming, two 1.0 mA 5 s scrambled footshocks, and 60 min of restraint. Swim and footshock stressors are repeated during the 7-day paradigm (Hammack et al., 2009; King et al., 2017) (**Figure 4A**). When compared with other animal models of stress (e.g., resident intruder, immobilization, WAS, and electrical footshock) where the same stressors are presented daily, the RVS paradigm is unique in that various stressors are presented throughout the 7-day protocol, which may be more relevant to human daily life stressors. Other advantages of the RVS paradigm include: (1) lack of habituation because of novel stressor exposure; (2) consistent and robust changes in urinary bladder function (Merrill et al., 2013b; Merrill and Vizzard, 2014) (**Figure 4D**) and (3) reproducible decrease (10%) in weight gain during RVS (Merrill et al., 2013b; Merrill and Vizzard, 2014) (**Figures 4B,C**). Furthermore, RVS paradigms are widely used to characterize the chronic stress response, including: (1) CNS and PNS responses and neurochemical plasticity to chronic stress; (2) comorbidity of stress-related disorders; and (3) the role of the limbic system and neuroendocrine cascade in chronic stress (Herman et al., 1996, 2003, 2005, 2012, 2016; Jankord and Herman, 2008; Jeanneteau et al., 2012). We recently extended the use of the RVS protocol to characterize the effects on the autonomic nervous system and somatic sensitivity (Merrill et al., 2013b; Merrill and Vizzard, 2014). RVS produced (1) a decrease in bladder capacity and void volume and an increase in voiding frequency (**Figure 4D**); (2) enhanced referred somatic sensitivity of both the hindpaw and pelvic regions; and (3) changes in the inflammatory milieu (e.g., histamine, myeloperoxidase, NGF, CXCL12) of the urinary bladder (Merrill et al., 2013b; Merrill and Vizzard, 2014).

## RVS and PACAP/Receptor Mechanisms Contribute to Micturition Dysfunction

**FIGURE 5:**
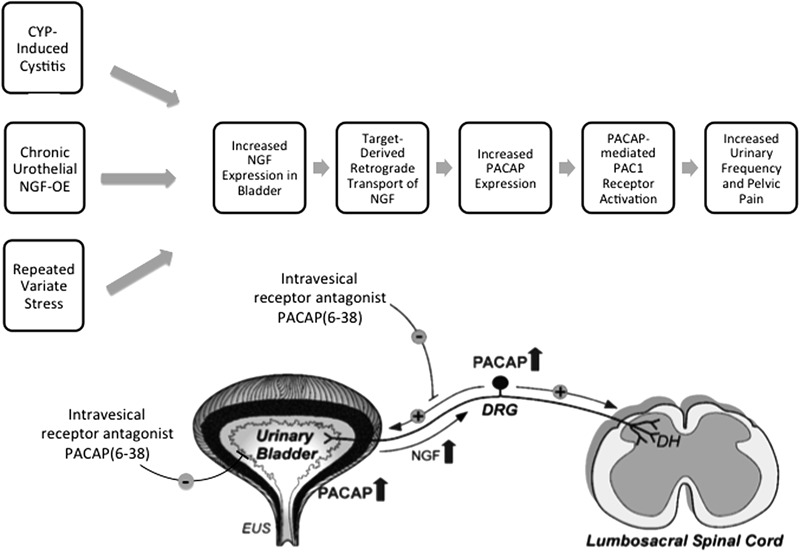
The PACAP/receptor system in micturition reflexes: a potential target for therapeutic intervention. CYP-induced bladder inflammation model, the transgenic mouse model of chronic urothelial NGF-OE and a repeated variate stress model are useful to evaluate underlying mechanisms contributing to urinary bladder dysfunction and pelvic pain. Each model is associated with changes in NGF expression in the urinary bladder that can result in changes in the urinary bladder and be transported in a retrograde manner to lumbosacral dorsal root ganglia (DRG). PACAP and PAC1 receptor exhibit neuroplastic changes in expression and function with bladder inflammation, and stress. Intravesical instillation of the PAC1 receptor antagonist, PACAP(6-38), reduces voiding frequency and somatic sensitivity in preclinical models. The PACAP/receptor system in micturition reflexes may represent a potential target for therapeutic intervention to improve urinary bladder function and reduce pelvic pain. DH, dorsal horn; EUS, external urethral sphincter. Figure modified from Braas et al. (2006).

Symptom exacerbation due to stress is prevalent in many disease states, including functional disorders of the urinary bladder (e.g., OAB, PBS/IC) and may be partly due to disruption of the HPA axis (Westropp and Buffington, 2002; Nazif et al., 2007). The prevalence of micturition disorders is high among individuals with anxiety disorders (Perry et al., 2006; Fan et al., 2008; Coyne et al., 2009). However, the mechanisms underlying the effects of stress on micturition reflex function are unclear. Among central neuroregulators, PACAP (*Adcyap1*) and PAC1 receptor (*Adcyap1r1*) are novel stress mediators (Dore et al., 2013). PACAP/PAC1 receptor transcripts are increased in specific limbic structures following RVS exposure; CNS PACAP signaling is anxiogenic and anorexic, and chronic stress-induced anxiety-related behaviors can be blocked by PAC1 receptor antagonists (Hammack et al., 2009; Lezak et al., 2014a,b; Missig et al., 2014; Roman et al., 2014). We have recently characterized PACAP/PAC1 signaling in stress-induced urinary bladder dysfunction in mice (Gonzalez et al., 2016; May et al., 2017b). RVS induced urinary bladder hyperreflexia characterized by reduced void volumes, increased voiding frequency and decreased intercontraction void intervals (Merrill et al., 2013b; Merrill and Vizzard, 2014). In studies using a resident intruder paradigm in mice, similar changes in urinary bladder function (i.e., increased voiding frequency) have also been demonstrated (Mingin et al., 2014, 2015). Plasma corticosterone, a steroid hormone involved in the HPA axis secreted by the adrenal gland in rodents during stressor exposure, was determined in RVS and control mouse groups. Plasma corticosterone was significantly increased (5.2-fold) in mice with RVS compared to control mice (May et al., 2017b). We determined PACAP and PAC1 transcript and protein expression in the urinary bladder and lumbosacral DRG and spinal cord in RVS or control mouse groups. PACAP mRNA was significantly increased in lumbosacral (L1, L2, L6, S1) DRG following RVS but no changes were observed in PACAP protein. PACAP protein was significantly increased in urinary bladder and in the lumbosacral spinal cord (L6, S1) following RVS. No changes were observed in PAC1 mRNA expression in lumbosacral DRG examined; however, PAC1 protein was significantly increased in L6 an S1 DRG. Bladder function was assessed in RVS and control mouse groups using continuous, intravesical infusion of saline in conscious, unrestrained mice with an open outlet both before and after PAC1 blockade at the level of the urinary bladder (Gonzalez et al., 2016; May et al., 2017b). Intravesical administration of the PAC1 receptor antagonist, PACAP(6-38) (300 nM), significantly increased intercontraction interval (2.5-fold) and void volume (2.5-fold) in mice following RVS (Gonzalez et al., 2016; May et al., 2017b). We also evaluated the effect of PAC1 blockade at the level of the urinary bladder on pelvic and hindpaw sensitivity in RVS or control mouse groups using von Frey filament testing. Intravesical administration of PACAP(6-38) (300 nM) significantly reduced pelvic and hindpaw sensitivity in mice following RVS (Gonzalez et al., 2016; May et al., 2017b). PACAP/receptor signaling contributes to the increased voiding frequency and pelvic and hindpaw sensitivity observed in mice following RVS (**Figure 5**) (Braas et al., 2006). Ongoing studies are evaluating the potential overlap of CNS and PNS pathways involved in micturition reflex function and stress responses.

## Perspectives and Significance

Numerous neuropeptide/receptor systems are expressed in CNS and PNS pathways that coordinate LUT reflexes [for review, see (Arms and Vizzard, 2011)]. PACAP is a member of the VIP/secretin/glucagon family of hormones, that is highly conserved across species and exists in two different isoforms, PACAP27 and PACAP38; the latter being the predominant form in most tissues and organ systems (Fahrenkrug et al., 1989; Arimura et al., 1991; Hannibal et al., 1995; Arimura, 1998; Fahrenkrug and Hannibal, 1998a,b). PACAP/receptor signaling underlies diverse neurological and physiological functions. Fahrenkrug and Hannibal (1998b) demonstrated that PACAP was expressed in C-fiber, unmyelinated nerve fibers in rat urinary tract. Given the involvement of C-fiber nerve pathways in the LUT following inflammation and neural injury (Fowler et al., 2008), we began to evaluate the anatomical distribution and function of PACAP/receptor signaling in rodent LUT pathways. PACAP (*Adcyap1*) and its cognate receptor, PAC1 (*Adcyap1r1*), have tissue-specific distributions in diverse systems including the LUT in both neural and non-neural components. Preclinical animal models including the CYP-induced bladder inflammation model, the transgenic mouse model of chronic urothelial NGF-OE and a RVS model are useful to evaluate underlying mechanisms contributing to urinary bladder dysfunction and pelvic pain associated with the clinical condition of PBS/IC (**Figure 5**). PACAP and associated receptors exhibit neuroplastic changes in expression and function with bladder inflammation or psychogenic stress and the PAC1 antagonist, PACAP(6-38), improves bladder function and reduces somatic sensitivity in preclinical models (**Figure 5**) (Braas et al., 2006). Changes in the PACAP/receptor system in micturition pathways may underlie and/or contribute to LUT dysfunction including the symptoms of increased voiding frequency, and pelvic pain. The PACAP/receptor system in micturition reflexes may represent a potential target for therapeutic intervention (**Figure 5**) (Braas et al., 2006).

We now draw attention to several areas where additional research is needed to advance our understanding of LUT function and dysfunction:

### The Need for Novel LUT Targets

This review with its focus on the PACAP/receptor system in micturition pathways and its potential as a novel target to improve bladder function, underscores the need for additional LUT targets for intervention. Given the heterogeneity of PBS/IC syndrome, it is unlikely that one targeted intervention will work for all.

### The Need for Preclinical Models

There are many challenges in identifying potential LUT targets. One challenge that continues to limit the identification and effectiveness of targets concerns the use of preclinical models and their relevance to human disease. For example, there is no consensus concerning the etiology of the disease syndrome, PBS/IC, which hinders the development and acceptance by the research community of preclinical models of PBS/IC (Lavelle et al., 2000; Sant and Hanno, 2001; Nazif et al., 2007; Quillin and Erickson, 2012; Saban, 2015). The models we have described in this review are several used to test hypotheses pertaining to LUT function and dysfunction. For example, the CYP model of bladder inflammation has grown in popularity and acceptance in the research community over the past 20 years. However, we still exercise caution and never consider the CYP model, a preclinical model of PBS/IC. Rather, CYP-induced cystitis is a bladder inflammation model that mirrors some signs and symptoms of the PBS/IC. The translational relevance of targets identified in preclinical models must be demonstrated in clinical studies.

### The Contribution of Stress in Bladder Dysfunction

As discussed in this review, BPS/IC symptoms are often exacerbated by stress and correlate with symptom severity; however, cause and effect have not been addressed (Rothrock et al., 2001a,b; Birder and Andersson, 2013). There are a growing number of preclinical stress models being used to determine underlying mechanisms of stress-induced urinary bladder dysfunction (Miczek, 1979; Imaki et al., 1991; Spanos et al., 1997; Alexacos et al., 1999; Boucher et al., 2002, 2010; Cetinel et al., 2005; Bhatnagar et al., 2006; Robbins et al., 2007; Robbins and Ness, 2008; Black et al., 2009; Chang et al., 2009; Wood et al., 2009; Smith et al., 2011; Merrill et al., 2013b; Merrill and Vizzard, 2014). As an important first step, determining the overlap in the PNS and CNS neural structures and pathways that underlie stress responses and micturition reflexes will provide insight into the anatomical substrates underlying these processes. These studies should be followed by studies determining the neurochemical phenotype of participating cells in identified PNS and CNS substrates.

### The Function of the Lamina Propria Signaling Network

Additional attention should be focused on unexplored and unappreciated aspects of the urinary bladder structure that could have profound influence on bladder function. As described previously, the lamina propria is located between the basement membrane of the mucosa and the detrusor muscle and is composed several types of cells, including: fibroblasts, adipocytes, interstitial cells, and afferent and efferent nerve endings (Andersson and McCloskey, 2014). Although increasing attention is being focused on the lamina propria, its contribution to bladder function is still emerging (McCloskey, 2010; Johnston et al., 2012; Andersson and McCloskey, 2014). We have recently observed slow propagating waves of activity in the lamina propria network that displayed varying degrees of coupling (Heppner et al., 2017). Application of ATP or TRPV4 agonist, GSK1016790 (100 nM), increased the duration of Ca^2+^ events, the number of cells with Ca^2+^ events and the integrated Ca^2+^ activity corresponding to propagation of activity among cells in the lamina propria network (Heppner et al., 2017). These findings indicate that ATP and TRPV4 can activate cells in the laminar propria network, leading to the appearance of organized propagating wavefronts (Heppner et al., 2017). Continued analyses of the lamina propria network should include determining if neuropeptide/receptor systems can also activate cells in the lamina propria network and lead to propagating wavefronts. Such studies will help in understanding the potential functional importance of the lamina propria in health and disease.

## Studies Involving Animal Research

The studies described from the Vizzard laboratory were performed in accordance with institutional and national guidelines and regulations. The University of Vermont Institutional Animal Care and Use Committee approved all experimental protocols involving animal use. Animal care was under the supervision of the University of Vermont’s Office of Animal Care Management in accordance with the Association for Assessment and Accreditation of Laboratory Animal Care (AAALAC) and National Institutes of Health guidelines. All efforts were made to minimize the potential for animal pain, stress or distress.

## Author Contributions

Conceived, discussed, and outlined the review: BG, KT, and MV. Reviewed the literature cited: BG, KT, and MV. Drafted and revised paper: BG, KT, and MV. Wrote the paper: BG, KT, and MV.

## Conflict of Interest Statement

The authors declare that the research was conducted in the absence of any commercial or financial relationships that could be construed as a potential conflict of interest.

## Abbreviations

AC: adenylate cyclase
ATP: adenosine triphosphate
B1: bradykinin receptor B1
B2: bradykinin receptor B2
BAC: bacterial artificial chromosome
BDNF: brain-derived neurotrophic factor
BNST: bed nucleus of the stria terminalis
BOO: bladder outlet obstruction
cAMP: cyclic adenosine monophosphate
CGRP: calcitonin gene-related peptide
CNS: central nervous system
CRH: corticotropin releasing hormone
CYP: cyclophosphamide
DH: dorsal horn
DRG: dorsal root ganglia
FIC: feline interstitial cystitis
FKBP5: FK506-binding protein 5
HPA: hypothalamic–pituitary–adrenal
IR: immunoreactivity
LCP: lateral collateral pathway
LUT: lower urinary tract
MPG: major pelvic ganglia
mRNA: messenger ribonucleic acid
NGF: nerve growth factor
NGF-OE: nerve growth factor overexpression
NO: nitric oxide
OAB: overactive bladder
P2X and P2Y: purinergic receptors
p75^NTR^: p75 neurotrophin receptor
PAC1: PACAP receptor 1
PACAP: pituitary adenylate cyclase-activating polypeptide
PACAP-EGFP: PACAP-enhanced green fluorescent protein
PAG: periaqueductal gray
PBS/IC: painful bladder syndrome/interstitial cystitis
PC: pheochromocytoma
PCR: polymerase chain reaction
PLC: phospholipase C
PMC: pontine micturition center
PNS: peripheral nervous system
PTSD: post-traumatic stress disorder
RVS: repeated variate stress
SCI: spinal cord injury
SP: substance P
SPN: sacral parasympathetic nucleus
TrkA: tropomyosin receptor kinase A
TrkB: tropomyosin receptor kinase B
TRPA1: transient receptor potential ankyrin 1
TRPM8: transient receptor potential melastatin 8
TRPV1: transient receptor potential vanilloid 1
TRPV2: transient receptor potential vanilloid 2
TRPV4: transient receptor potential vanilloid 4
VIP: vasoactive intestinal polypeptide
VPAC1: VIP receptor 1
VPAC2: VIP receptor 2
WAS: water avoidance stress
WT: wildtype
